# New Departures in Diagnosis

**Published:** 1886-03

**Authors:** 


### NEW DEPARTURES IN DIAGNOSIS.

The principles which should control the surgeon and the physician in diagnosticating diseases look to the exercise of those faculties of discrimination by our senses and intellects that are based upon a comprehension of all the surroundings of the case. A thorough study of the physical signs of disease in the organism, assisted by manual and instrumental exploration, has heretofore been considered as requisite to forming a correct judgment as to the site and nature of any disorder; and great painstaking in obscure cases has been commended in resorting to chemical tests, electrical appliances and microscopical examinations for verifying the accuracy of the diagnosis made by the ordinary physical and rational proceedings. In view of these well-recognized facts, it must strike the enlightened members of the medical profession in America and Great Britain as a great departure from the orthodox standard for the investigation of disease, that two very eminent practitioners in England should have ignored such preconceived guides in setting up their individual claims to a species of intuition in determining upon the distinguishing features of morbid phenomena in special departments of diagnosis.

In his first Lettsomian lecture, Mr. Jonathan Hutchinson remarks that "our rules of diagnosis have been, I cannot but think, far too definitely laid down on these matters," referring to the distinguishing signs of chancroid and chancre. His whole course of reasoning tends to obfuscate the effort at reaching a distinct perception of the characteristics of these two phases of venereal diseases; and if any final conclusion is to be drawn from his argumentation, it is that the so-called chancroid should be classed with phagedemic ulceration of a non-specific nature. It matters not practically what this disease may be styled, if it is admitted to be incapable of producing constitutional effects, and this is all that is claimed by the dual syphilitic theory. But with the very high reputation of the author for discriminating judgment, all must still be impressed with the inadequacy of his opinions to overturn the testimony of other observers.

If we turn our attention to the paper of Mr. Lawson Tait, on "Methods of Diagnosis," presented to the recent meeting of the Medical Society of the State of New York, it is stated, in speaking of the use of the speculum and sound to obtain accurate information as to the conditions of the vaginal mucous surface of the os and cervix, and as to the position of the fundus or the relations of the uterus, that "no practitioner of gynæcology can possibly be regarded, at least by me, as an accomplished specialist, who uses either one or the other of these instruments with great frequency." "As a matter of fact, I have found that these two instruments, the speculum and sound, as methods of diagnosis, have been productive of uniformly more harm than good." And to sustain his position, he repeats a story of having caused a premature birth by passing a sound into a young woman's uterus, who was four months advanced in pregnancy, under the impression that the patient was suffering from a perimetric abscess, and professes to have thereby learned a useful lesson, of which at the time he stood very much in need. He concludes that because the improper employment of the sound is attended with serious consequences, "the sound is one of the most dangerous instruments which ever were invented for the treatment of human suffering," without referring to the advantages of resorting to this mode of exploring and reducing conditions of the womb to which it is applicable. Such is a fair sample, given in Mr. Tait's own words, of his manner of dealing with many recognized measures for diagnosticating uterine and other disorders of the abdominal cavity.

In lieu of orthodox precedents, he is unable to describe how he reaches his conclusions, saying, in one case, with an air of great frankness, "but I know that my educated finger-tips can make this distinction;" and after condemning the inexperienced hands in another state of things, remarks that "the gentlest and tenderest touch alone will reveal what is required."

After reading carefully this narration of the slight of hand practiced by Mr. Tait, we may commend to him the appositeness of the words—

> "Wad some power the giftie gie us,
> To see oursels as others see us;
> It wad frae mony a blunder free us,
> And foolish notion," etc.

"It has been my misfortune," says Mr. Lawson Tait, "never to have been a teacher," and the reader will naturally conclude that it may be his good fortune never to become a teacher of others in the art of diagnosis, as woe be unto the unlucky gynæcologist who undertakes to throw aside the great landmarks for distinguishing morbid developments for the adoption of his new doctrine of "the rule of thumb" inculcated in this paper on methods of diagnosis.

While claiming that inspection is one of the most important methods of diagnosis in abdominal disease, concerning which a very great deal of nonsense has been talked, he makes his customary fling at Sir Spencer Wells for holding that inspection will reveal the presence or absence of adhesions. But in regard to other abnormal developments, Mr. Lawson Tait allows that "a careful examination, by the eye, of the *contour* of the abdomen, when the patient is lying on her back, with the walls of the abdomen perfectly flaccid, will reveal a good deal to the experienced practitioner." "The process of discriminating between these various conditions may very rapidly be completed by one who is accustomed to dealing with them," as is Mr. Tait. His prestidigitation is only equalled by his optical skill. "Thus peritonitis may be at once detected or eliminated by the presence or absence of the short and rapid pectoral breathing which shows that the patient is loth to use her diaphragm. In fact, by this alone, and without almost any further inquiry, I have satisfied myself as to the nature of the case by a single glance."

His methods of diagnosis are clearly "springs to catch woodcocks," and it is not likely that many on this side of the water will fall into the snare. But while these precepts are misleading, the results of Mr. Tait are worthy of commendation.

### ITEMS.

Dr. D. H. HOWELL, of this city, has recently returned from a trip to Cuba.

THE Alabama State Medical Association will meet in Anniston, April 13.

DR. A. W. GRIGGS, a prominent physician of West Point, was in the city a few days since.

THE THIRTY-SEVENTH Annual Session of the American Medical Association will be held in St. Louis, Mo., on Tuesday, Wednesday, Thursday and Friday, May 4, 5, 6 and 7, commencing on Tuesday at 11 A. M.

TRYPSIN AS A SOLVENT FOR DIPHTHERITIC MEMBRANE.—The well-known properties of this principle of the pancreatic juice, manufactured by Fairchild Bros. & Foster, give the strongest grounds for anticipating success in its application for this important purpose.

Trypsin acts quickly and powerfully upon fibrin and fibrinous membrane. It is not dependent upon the inter-action of acid as is the case with pepsine. It is most active in a slightly alkaline media. It may be applied by spray or brush. In practical use the results have been very encouraging.

WE recently had a pleasant call from Dr. R. O. Cotter, of Macon. Dr. Cotter was for several years associated with Dr. A. W. Calhoun as assistant to the chair of diseases of eye, ear and throat in the Atlanta Medical College. Dr. Cotter is Secretary of the Macon Medical Society. His friends will be glad to know that he is meeting with success as an oculist and aurist in the Central City.

NEW FRENCH JOURNALS.—Two new gynæcological journals have appeared in Paris—the *Nouvelles Archives d' obstetrique ct de Gynecologie*, issued monthly, and the *Repertoire Universel d' obstetrique et de Gynecologie*, issued semi-monthly. Both journals are under the same management and are edited by Charpentier, Duplay, Bernutz and other leaders of gynæcology in France.

RETAINED MENSES FROM ATRESIA OF OS UTERI.—In the *Journal de Medecine* of February 7 is reported a case of retained menses in a girl of fourteen from atresia of the os uteri. The distended womb formed a tumor as large as that of the ninth month of pregnancy. As remarked by the reporter, M. Després, tumors of such size from menstrual retention are rare at so early an age.

MR. JOHN W. COX, of Boston, representing Doliber, Goodale & Co., the manufacturers of Mellin's food for infants and invalids, gave us a call a few days since. He will call on the physicians in Georgia in the interest of this food. From our knowledge of it, and the experience we have had with it, extending over a period of several years, we feel no hesitancy in recommending it to the favorable consideration of our readers.

URETHANE, THE NEW HYPNOTIC.—At a recent meeting of the *Societe de Therapeutique* of Paris (*Le Progres Medical*), M. Huchard reported the results of his investigations of *urethane*, the new hypnotic. This remedy exists in the form of white crystals of a faint odor and peculiar taste. It is very soluble in water, alcohol and ether. The dose for an adult is from 2 to 3½ grammes (30 to 60 grains). Its effect is claimed to be in all respects similar to physiological sleep. It may be safely given to infants in doses of 20 centi-grammes (3 grains) in orange flower water or simple syrup.

AIR-EMBOLISM.—We have received a paper on this subject from the author, Dr. N. Senn, Professor of Surgery in the College of Physicians and Surgeons, Chicago, Ill., reprint from the Transactions of the American Surgical Association of 1885.

A most onerous task has been well executed by Prof. Senn. This is a neatly printed octavo pamphlet of 121 pages, exhibiting great research in the collection of data from other sources and much care and diligence in the performance of experiments upon inferior animals. The very important precautionary measures against air-embolism in case a vein is wounded in the "danger zone" are entitled to the serious consideration of all operators under the divisions of position, compression, ligature and aseptic tampon. The following therapeutic indications should be met promptly: Prevention of further ingress of air, use of cardiac stimulants, venesection, aspiration of air from the right side of the heart, as "desperate cases call for desperate measures."

OIL OF TURPENTINE IN DIPHTHERIA.—In the *Journal de Medecine* of January 31, Dr. Delthil, of Paris, reports a case of diphtheria in a child thirty-two months old, in which the patient occupied the same room with four puerperal women and their newly-born infants. Circumstances rendered isolation impossible. The whole mucous membrane of the patient from the lips to the bronchi, including the nasal passages, was covered with a thick diphtheritic exudation. The treatment consisted of quinine, emetics, abundant nourishment and painting the affected mucous membrane with undiluted oil of turpentine every hour. To protect the puerperal women and their children from contagion, the atmosphere of the room was kept saturated with vapor produced by the evaporation of oil of turpentine and fumes from the combustion of a mixture of oil of turpentine and coal tar. The patient recovered, and neither the puerperal women nor their newly-born infants were infected. To facilitate applications to the throats of young children, the writer recommends the novel expedient of tickling the fauces with a feather introduced between the teeth. The efforts at vomiting excited thereby will cause the child to open its mouth and the application may then be easily made.

S. CECCHINI (Italy) reports (*Centralblatt f. Chirurg., Jan. 2, 1886*) on the radical cure of anal and dental fistula, also fistula of Steno's duct and caries of the petrous portion of the temporal bone by means of injection with oil of turpentine, diluted, when necessary, with almond or olive oil. The action of the turpentine is supposed to be as an active stimulant to the granulation and as an anti-mycotic. Many cases are presented in which complete cure was effected.

WE observe that Dr. Piffard has retired from his editorial connection with the *Journal of Cutaneous and Venereal Diseases.* The Journal will be continued under the sole editorial charge of Dr. P. A. Morrow. We may remind our readers that this is the only publication in the English language devoted to skin and venereal diseases, and, during the three years of its existence, it has won for itself a high reputation for scientific excellence as well as practical utility. In addition to presenting all that is new and valuable in these special departments, the colored lithographs and wood engravings with which the original articles are illustrated are worth more than the price of subscription.

A CORRECTION.—In the February issue of THE JOURNAL, page 749, second line from top, for eczema *fissures* read eczema *fissum.*

CATARRH OF THE BLADDER.—A writer in the London *Lancet*, of October 31, 1885, reports two cases of catarrh of the bladder treated with Parke, Davis & Co.'s fluid extract of corn-silk with excellent results.

MRS. FRANCES H. BURNETT, the novelist, has written a serial story for *St. Nicholas*, called "Little Lord Fauntleroy," the hero of which is a boy-character who is as new as he is delightful. The story was begun in the present volume of *St. Nicholas* and will run through the year. Mrs. Burnett is at work on a new novel for *The Century*.

"THE Feeding of Infants" is the title of an article by Dr. J. Lewis Smith, in *Babyhood* for January, in which the writer graphically describes certain evil effects of artificial feeding, and gives important directions for weaning and nursing. Among other articles are: "The Baby's Mother," by Helen P. Grafton; "Our Baby and How we Undid Her" (the first of a humorous series), by Martha O. Inglis; "Cat-Naps and their Causes," "Baby's Curls," etc.

MONTHLY METEOROLOGICAL SUMMARY, ATLANTA, FOR JANUARY, 1886.

| | |
|---|---|
| Mean Temperature, | 36.1 |
| Highest Temperature, January 3rd, | 59.9 |
| Lowest Temperature, January 11th, below zero, | 2.4 |
| Monthly Range of Temperature, | 62.3 |
| Greatest Daily Range of Temperature, | 29.0 |
| Least Daily Range of Temperature, | 6.8 |
| Mean Daily Range of Temperature, | 16.1 |
| Prevailing Direction of Wind, | N. W. |
| Total Movement of Wind, | 8,857 Miles. |
| Highest Velocity of Wind and Direction, | 36 Miles, S. E. and W. |
| No. of Days on which .01 inch or more of Rain or Snow fell, | 13 |
| Depth of unmelted Snow on ground at end of month | 0 inches |
| Number of Foggy Days, | None. |
| Number of Clear Days, | 9 |
| Number of Fair Days, | 10 |
| Number of Cloudy Days, | 12 |
| Dates of Frosts { Light, | 6, 7, 18, 20. |
| Dates of Frosts { Killing, | 9, 10, 11, 12, 13, 14, 17, 22, 30. |
| Dates of Thunder-storms, | 20th. |

